# Aging of gray matter microstructure: A brain-wide characterization of age group differences using NODDI

**DOI:** 10.1016/j.neurobiolaging.2025.02.004

**Published:** 2025-02-19

**Authors:** Danielle Greenman, Ilana J. Bennett

**Affiliations:** Department of Psychology, University of California, Riverside, USA

**Keywords:** Normal aging, Diffusion MRI, NODDI, Gray matter, Cortex, Hippocampus, Striatum, Atrophy, Inflammation, Iron

## Abstract

This study aimed to provide a complete characterization of age group differences in cortical lobar, hippocampal, and subcortical gray matter microstructure using a multi-compartment diffusion-weighted MRI (DWI) approach with parameters optimized for gray matter (Neurite Orientation Dispersion and Density Imaging, NODDI). 76 younger (undergraduate students) and 64 older (surrounding communities) adults underwent diffusion-, T1-, and susceptibility-weighted MRI. Results revealed eight unique patterns across the 12 regions of interest in the relative direction and magnitude of age effects across NODDI metrics, which were grouped into three prominent patterns: cortical gray matter had predominantly higher free diffusion in older than younger adults, the hippocampus and amygdala had predominantly higher dispersion of diffusion and intracellular diffusion in older than younger adults, and the putamen and globus pallidus had lower dispersion of diffusion in older than younger adults. Results remained largely unchanged after controlling for normalized regional volume, suggesting that higher free diffusion in older than younger adults in cortical gray matter was not driven by macrostructural atrophy. Results also remained largely unchanged after controlling for iron content (QSM, R_2_*), even in iron-rich subcortical regions. Taken together, these patterns of age effects across NODDI metrics provide evidence of region-specific neurobiological substrates of aging of gray matter microstructure.

## 1. Introduction

Magnetic resonance imaging (MRI) has been a critical tool for studying effects of aging on the brain *in vivo*, demonstrating that different brain regions are impacted by normal aging and early pathological processes to varying degrees. For example, T1-weighted MRI has revealed that the aging brain atrophies, with tissue loss being greatest in frontal and temporal cortex and in the hippocampus, nucleus accumbens, and thalamus (Fjell et al., 2013; Fjell and Walhovd, 2010; Long et al., 2012; Raz et al., 2010). Susceptibility-weighted MRI has shown that iron accumulates with age in the globus pallidus and putamen, whereas cortical gray matter and the hippocampus are relatively spared (Daugherty and Raz, 2013; Ficiarà et al., 2022; Guerreri et al., 2019; Haacke et al., 2005). Diffusion-weighted MRI (DWI) has revealed age-related degradation of tissue microstructure that is prominent in frontal white matter, consistent with demyelination (Bennett et al., 2010; Madden et al., 2012; Mendez Colmenares et al., 2023), with an emerging line of research suggesting that more advanced (multi--compartment) DWI models may be sensitive to gliosis and neurodegeneration that accompany neuroinflammation in gray matter (Garcia-Hernandez et al., 2022; Radhakrishnan et al., 2022b; Venkatesh et al., 2021). However, a complete characterization of the region-specificity of age group differences in brain-wide gray matter microstructure using advanced DWI models is currently lacking.

DWI evaluates tissue microstructure by measuring the movement of molecular water and then estimating various properties of the measured diffusion, which can be modelled using either single-tensor (Basser et al., 1994) or a variety of multi-compartment (e.g., Assaf et al., 2008; Assaf and Basser, 2005; Fieremans et al., 2011; Jelescu et al., 2022; Zhang et al., 2012) algorithms. Neurite Orientation Dispersion and Density Imaging (NODDI; Zhang et al., 2012), for example, separately models the water movement that is highly restricted within small cell membranes as a set of sticks (*intracellular diffusion*; also known as the fraction of intracellular diffusion, fICVF), hindered between cells and within large cell somas as the spreading of the sticks (*dispersion of diffusion*; also known as the orientation dispersion index, ODI), and unrestricted outside of the cells as an isotropic sphere (*free diffusion*; also known as the fraction of isotropic diffusion, fISO). The default intrinsic diffusivity parameter can be adjusted to more accurately model the tissue compartments in gray matter (Fukutomi et al., 2019, 2018).

Different patterns of age effects in terms of the significance and direction of age group differences across NODDI metrics have been observed in cortical, hippocampal, and subcortical gray matter, consistent with the notion that there are region-specific neurobiological substrates of gray matter aging. For cortical gray matter, NODDI studies with adults across the lifespan have consistently reported lower dispersion of diffusion in older than younger adults (Filip et al., 2023; Nazeri et al., 2015; Ota et al., 2017), with this age effect appearing stronger in the parietal lobe (Nazeri et al., 2015). Intracellular diffusion mostly showed no significant age effects in cortical gray matter (Nazeri et al., 2015; Ota et al., 2017), although one study found that it was higher in older than younger adults (Filip et al., 2023). Whereas free diffusion was not included in any of these lifespan studies, examinations limited to older adults have found that higher cortical free diffusion relates to older age (Anderson et al., 2024; Merluzzi et al., 2016; Yu et al., 2024). Thus, aging of cortical gray matter appears to be characterized by lower dispersion of diffusion and higher free diffusion. Importantly, however, none of these prior studies adjusted the default intrinsic diffusivity parameter to optimize NODDI estimates for gray matter, which can result in different NODDI values that vary with age (Guerrero et al., 2019).

For hippocampal gray matter, intracellular, dispersion, and free diffusion are consistently found to be higher in older than younger adults. Our group has reported this pattern of age effects for whole hippocampus (Franco et al., 2021; Ibrahim and Bennett, 2023; Venkatesh et al., 2021, 2020) and its subfields (Radhakrishnan et al., 2022a, 2020) using samples that at least partially overlap with those included in the current study. Lifespan NODDI studies similarly found higher dispersion (Metzler-Baddeley et al., 2019; Nazeri et al., 2015) and free (Metzler-Baddeley et al., 2019) diffusion in older than younger adults (c.f., Ota et al., 2017), but no significant age effect for intracellular diffusion. However, differences in the relative magnitude of age effects between NODDI metrics have not been directly tested, and this may be informative about underlying neurobiological substrates.

For subcortical gray matter, there is a dearth of literature examining age-related differences in NODDI metrics with the exception of dorsal striatum. For the caudate and putamen, intracellular and free diffusion are consistently found to be higher in older than younger adults when using region of interest approaches (Franco et al., 2021; Guerreri et al., 2019; Venkatesh et al., 2021), whereas dispersion of diffusion is lower in older than younger adults in some studies (Franco et al., 2021) and shows no significant age effect in others (Guerreri et al., 2019; Venkatesh et al., 2021). Voxel-wise approaches in lifespan samples have replicated these mixed age effects for caudate dispersion of diffusion (Nazeri et al., 2015; Ota et al., 2017), reporting no other significant age effects in any subcortical region, although they did not include free diffusion. For the globus pallidus, most studies find that intracellular diffusion is higher in older than younger adults, with no significant age effect for intracellular of free diffusion (Franco et al., 2021; Venkatesh et al., 2021;c.f., Guerreri et al., 2019). For the thalamus, just one study reported higher free and lower dispersion of diffusion in older than younger adults for thalamus, with no significant age effect for intracellular diffusion (Guerreri et al., 2019). For the nucleus accumbens, just one study found higher intracellular and free in older than younger adults, with no significant age effect for dispersion of diffusion (Franco et al., 2021). The amygdala, on the other hand, has not been included in any NODDI studies comparing younger and older adults. Thus, whereas aging of some subcortical gray matter is characterized by higher intracellular (globus pallidus) and free (caudate, putamen) diffusion, there is not sufficient evidence to draw conclusions about age effects in other regions (thalamus, nucleus accumbens, amygdala).

The current study aimed to provide a complete characterization of age group differences in gray matter microstructure that addressed gaps in prior research by examining all NODDI metrics and gray matter regions (cortical, hippocampal, subcortical) in the same sample of younger and older adults, using an advanced NODDI model with the intrinsic diffusivity parameter optimized for gray matter, and focusing on the relative magnitude of age group differences between NODDI metrics. We further aimed to extend prior work by separately controlling for T1-weighted MRI measures of volume and susceptibility-weighted MRI measures of iron content (QSM, R_2_*) in each region to examine the extent to which these neurobiological substrates contribute to age group differences in gray matter microstructure.

## 2. Methods

### 2.1. Participants

Younger adults were recruited via the University of California, Riverside Undergraduate Participant Pool (Sona Systems) and older adults (>65 years) were recruited from the surrounding communities via direct mailers and online ads. Participants were screened over the phone for physical, neurological, and MRI scanner contraindications. Of the 89 younger and 81 older adults who were enrolled in the study, we excluded seven with poor general cognition based on the Mini-Mental State Exam (MMSE > 27; Folstein et al., 1975) or Montreal Cognitive Assessment (MoCA > 23; Nasreddine et al., 2005) (4 younger, 3 older), six with missing MRI data (3 younger, 3 older), and 17 with anatomical (e.g., severe atrophy, lesions) or movement artifacts in their MRI data (6 younger, 11 older). One younger and one older adult were excluded from only the subcortical and cortical NODDI analyses, respectively, due to isolated MRI data issues (i.e., subcortical lesions, susceptibility artifacts). The final sample included 76 younger (18–29 years) and 64 older (65–87 years) adults. Demographic data is provided in Table 1.

All participants provided informed consent upon entry to the study and were compensated for their time, as approved by the University of California, Riverside Institutional Review Board.

### 2.2. MRI data acquisition

Imaging data were acquired using a 3 T Siemens Prisma MRI (Siemens Healthineers, Malvern, PA) scanner fitted with a 32-channel receive-only head coil. One high-resolution T1-weighted magnetization prepared rapid gradient-echo (MPRAGE) run was acquired with these parameters: echo time (TE)/repetition time (TR) = 2.72/2400 ms, voxel size = 0.8 mm^3^, 208 axial slices, and GRAPPA acceleration factor = 2.

Two diffusion-weighted echo-planar runs with opposite phase-encoding (anterior-to-posterior, posterior-to-anterior) were acquired with these parameters: TE/TR = 102/3500 ms, voxel size = 1.7 mm^3^, FOV = 212 × 182 mm, 64 axial slices, and multiband acceleration factor = 4. For each run, six volumes had no diffusion weighting (b = 0) and 128 volumes had bipolar diffusion gradients applied in 64 directions for each gradient strength (b = 1500 and 3000 s/mm^2^).

In a subset of participants (33 younger, 25 older), one 12-echo 3D gradient recalled echo (GRE) sequence was acquired using these parameters: TE/ΔTE/TR = 4/3/40 ms, voxel size = 1 × 1 × 1.7 mm, FOV = 192 × 224 × 163.2 mm, and GRAPPA acceleration factor = 2.

### 2.3. MRI data preprocessing

#### Region of Interest Segmentation.

Bilateral cortical regions of interest (frontal, insular, temporal, parietal, occipital) were segmented from the Montreal Neurological Institute probabilistic atlas (MNI-max-prob-thr0–1mm) in FSL (FMRIB Software Library; Jenkinson et al., 2012), thresholded at 10 % (zeroing probabilities below 0.1), and registered to each participant’s diffusion space (fractional anisotropy [FA] image) using the default combination of rigid, affine, and deformable syn transformations (*antsRegistrationSyN.sh*) with ANTs (Advanced Neuroimaging Tools; Avants et al., 2011). The diffusion space-aligned cortical masks were further limited to gray matter by excluding voxels containing white matter (FA > 0.15) and cerebrospinal fluid (non-tissue output of two tissue-type segmentation of the *b*=0 image). The diffusion space-aligned hippocampus mask (described next) was also excluded from the temporal lobar mask.

Bilateral hippocampus and subcortical regions of interest (amygdala, nucleus accumbens, thalamus, caudate, putamen, globus pallidum) were automatically segmented on each participant’s T1-weighted MPRAGE image using FSL’s *FIRST* (Patenaude et al., 2011), with the three-stage affine registration option for hippocampus, after it had been brain extracted using AFNI’s (Analysis of Functional NeuroImages; Cox, 1996) *3dSkullStrip*. For each region, segmented masks were binarized, combined across hemispheres, and then registered to each participant’s diffusion space (averaged *b*=0 image) using a rigid body transformation with a boundary-based registration (BBR; Greve and Fischl, 2009) cost function in FSL’s *FLIRT*.

#### Gray Matter Microstructure.

Diffusion-weighted images were preprocessed for each participant using FSL’s *topup* to correct for susceptibility-induced off-resonance fields (Andersson et al., 2003); *eddy* to correct for distortions due to eddy-currents, susceptibility, and motion (Andersson and Sotiropoulos, 2016); and *dtifit* to generate voxel-wise fractional anisotropy (FA) images for registrations and thresholding.

The NODDI MATLAB toolbox was used to estimate intracellular diffusion (fICVF), dispersion of diffusion (ODI), and free diffusion (fISO) metrics for each voxel, with the intrinsic diffusivity assumption set to 1.1 × 10^−3^ mm^2^/s to more accurately model the tissue compartments in gray matter (Fukutomi et al., 2019, 2018; Guerrero et al., 2019). All NODDI outputs were thresholded to exclude voxels with measurement artifacts (intracellular diffusion > 99 %) (Emmenegger et al., 2021) and tissue compartment outputs (intracellular, dispersion of diffusion) were thresholded to exclude voxels with low tissue content (free diffusion > 90 %) that may lead to unreliable estimates. For each participant, NODDI metrics were extracted from each region of interest by multiplying their diffusion space-aligned masks by the thresholded voxel-wise NODDI outputs.

#### Normalized volume.

Normalized volume of each region of interest was calculated from each participant’s T1-weighted MPRAGE image using a residual method to account for individual differences in brain size (Jack et al., 1989). Each participant had measures of volume in each region of interest (Region_Volume_raw_), as described above, and their intracranial volume (Intracranial_Volume_indiv_) was measured using the estimated total intracranial volume (eTIVindiv) generated by Freesurfer (Buckner et al., 2004). Mean intracranial volume (Intracranial_Volume_mean_) and the slope (β) of the relationship between intracranial volume and volume in each region of interest were calculated within younger adults only to get estimates of these measures in the absence of age-related atrophy. Then, for each participant and region, we applied the following equation: Region_Volume_norm_= Region_Volume_raw_ – β (Intracranial_Volume_indiv_ – Intracranial_Volume_mean_).

#### Iron content.

Phase and magnitude images from the 12-echo GRE sequence were used to calculate QSM and R_2_* metrics, respectively. Phase maps were unwrapped (Langley and Zhao, 2009), filtered using the spherical mean value method (Schweser et al., 2011), and then QSM values were estimated using an improved least-squares method (Li et al., 2015, 2011) and Laplace filtering. R_2_* values were estimated by fitting a monoexponential model to the GRE images. As in our prior work (Petok et al., 2024), we used relative QSM values with no reference given debates in the field regarding the appropriate reference region (usually ventricles or white matter) and known age effects on their underlying tissue properties (e.g., age-related demyelination and white matter lesions) that can bias the metrics (Deistung et al., 2017; Ravanfar et al., 2021). For each participant, mean QSM and R_2_* metrics were extracted from each region of interest after it was aligned to the iron maps using a transformation between their T1-weighted MPRAGE image and magnitude image from the first echo using FSL’s *FLIRT* and eroded by the default 3 mm^3^ box kernel in *fslmaths*.

### 2.4. Statistical approach

Given our interest in the relative magnitude of age group differences between NODDI metrics, primary analyses used separate omnibus 2 Age Group (younger, older) × 3 NODDI Metric (intracellular, dispersion, free) mixed factorial ANOVAs for each region of interest, with Age Group as a between-subjects measure and NODDI Metric as a within-subjects measure. Because the Metric variable has three levels, significant interactions in each region were further probed using three post hoc 2 Age Group x 2 Metric mixed factorial ANOVAs comparing each combination of NODDI metrics to identify which metrics showed significant differences in the magnitude of their age group difference and separate post hoc between-group *t*-tests for each Metric to identify which metrics showed significant age group differences.

Secondary analyses examined the influence of regional volume (normalized volume) and iron content (QSM, R_2_*) on the patterns of age group differences in gray matter microstructure. Age group differences in normalized volume and iron content were assessed in each region of interest using between-group *t*-tests. Separate 2 Age Group (younger, older) × 3 NODDI Metric (intracellular, dispersion, free) ANCOVAs were then conducted in each region controlling for either normalized volume or each iron metric in that region.

## 3. Results

### 3.1. Age group differences in gray matter microstructure by region

**Fig. 1.**
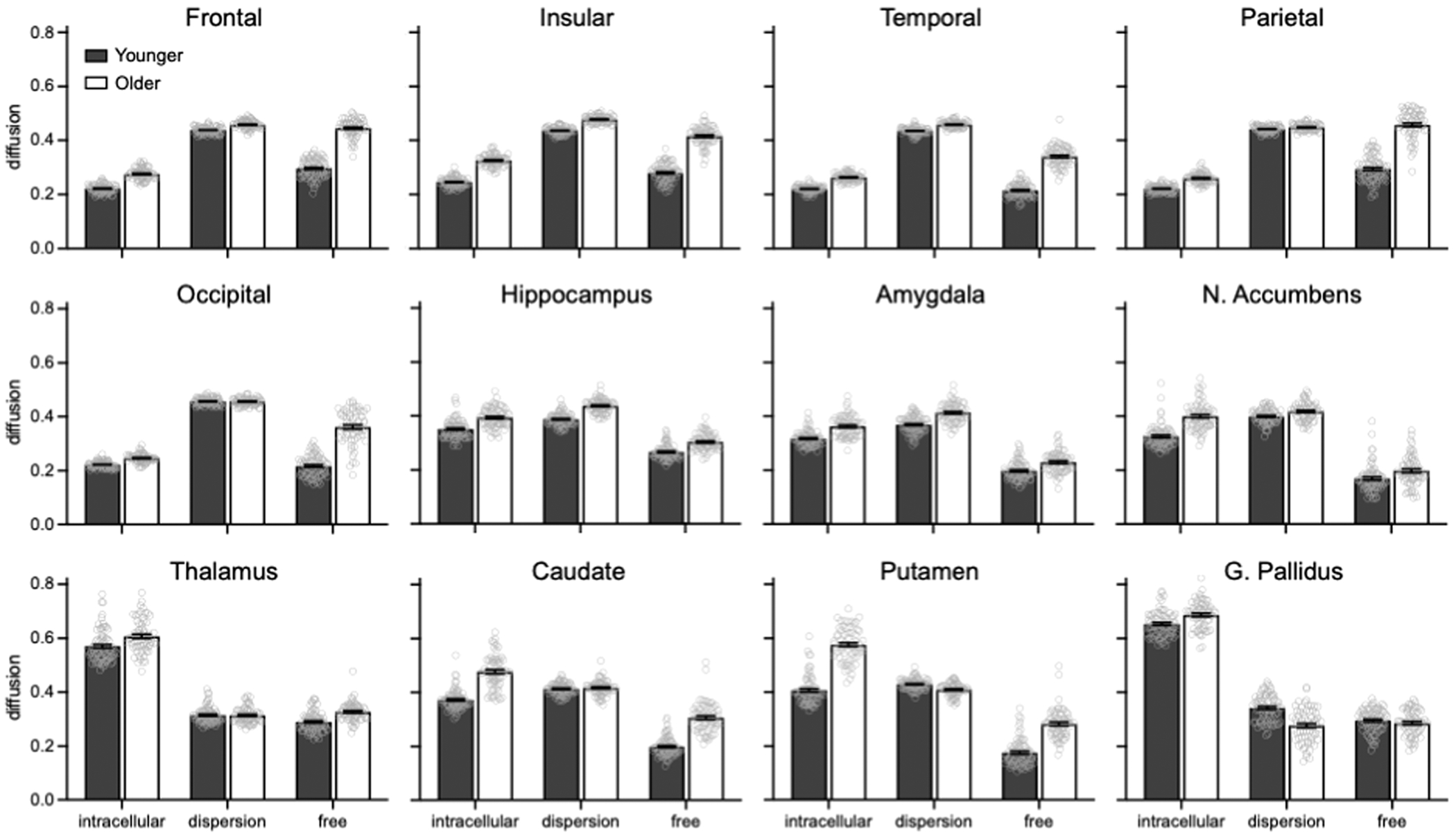
Bar graphs show differences between younger (gray bar) and older (white bar) adults for each NODDI metric (intracellular, dispersion, free) in each gray matter region of interest. Error bars denote standard error of the mean. Gray circles denote individual values.

**Fig. 2.**
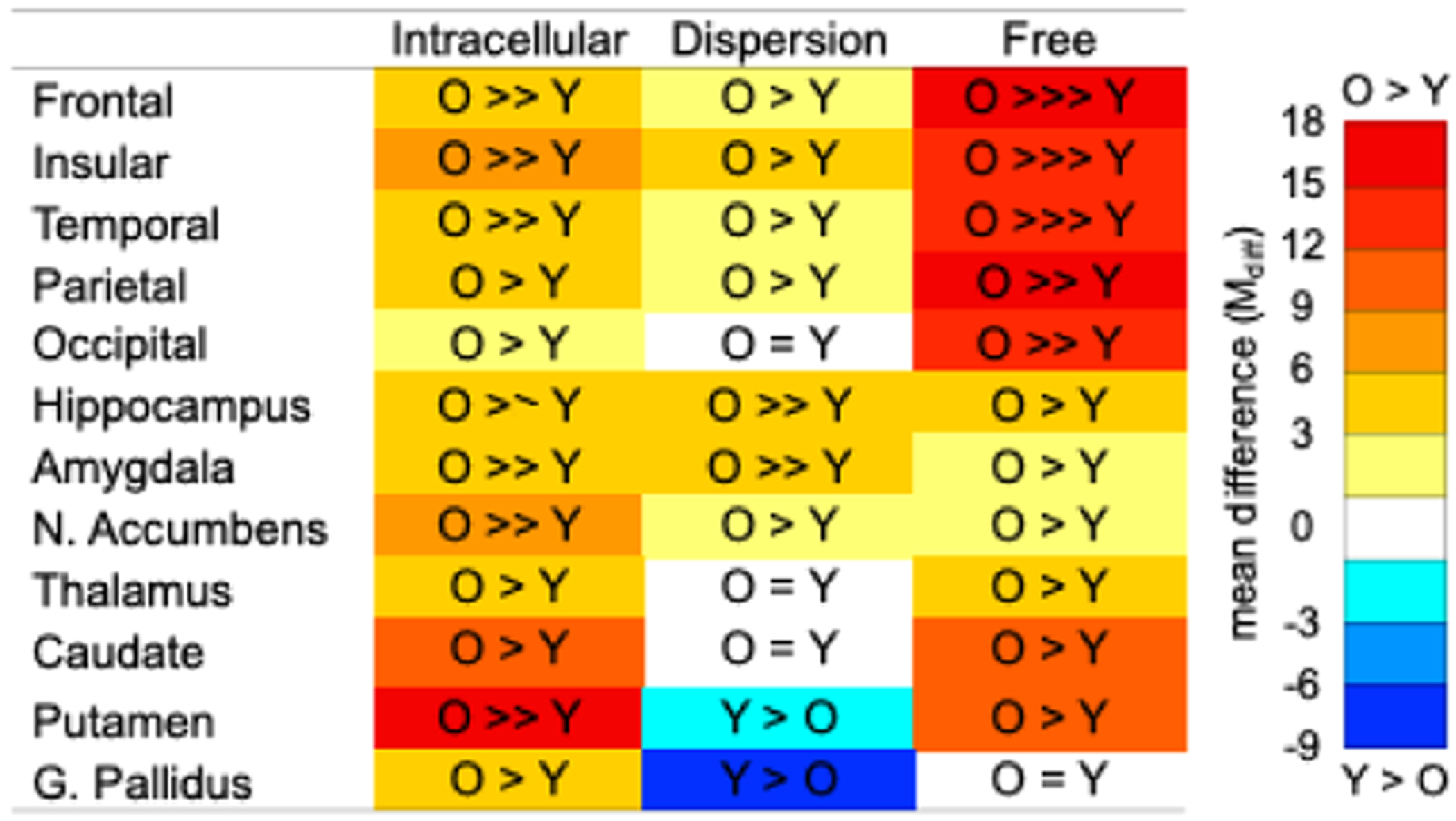
Age group differences between younger (Y) and older (O) adults are summarized for each NODDI metric and gray matter region of interest. Arrows indicate patterns of relative age group differences (>>> significantly largest, >> significantly larger, > significant, = not significant). For the hippocampus, a partial second arrow denotes that the age group difference for intracellular diffusion did not statistically differ from the other metrics. Colors provide a heatmap of the magnitude of age group differences.

Separate 2 Age Group × 3 NODDI Metric omnibus ANOVAs revealed significant effects of Age Group, Metric, and Age Group x Metric for each cortical (Table 2, top row) and subcortical (Table 3, top row) region of interest. Subsequent post hoc 2 Age Group x 2 Metric ANOVAs for each region (Supplementary Tables 1–2) and post hoc between-group *t*-tests for each Metric (Table 4) revealed eight unique patterns in the direction and magnitude of age effects across NODDI metrics, as detailed below. These data are presented in Fig. 1 and summarized in Fig. 2.

For frontal cortex, all diffusion metrics were significantly higher in older than younger adults, with the largest age group difference for free (mean difference: M_diff_ = 0.15), then intracellular (M_diff_ = 0.05), and then dispersion of diffusion (M_diff_ = 0.02). Insular (free M_diff_ = 0.14, intracellular M_diff_ = 0.08, dispersion M_diff_ = 0.04), temporal (free M_diff_ = 0.13, intracellular M_diff_ = 0.04, dispersion M_diff_ = 0.02), and parietal (free M_diff_ = 0.16, intracellular M_diff_ = 0.04, and dispersion M_diff_ = 0.01) cortex showed the same pattern of results.

For occipital cortex, free (M_diff_ = 0.14) and intracellular (M_diff_ = 0.02) diffusion were significantly higher in older than younger adults, with the largest age group difference for the former, whereas there was no significant age effect for dispersion of diffusion (M_diff_ = >−0.01).

For the hippocampus, all diffusion metrics were significantly higher in older than younger adults, with the largest age group difference for dispersion of diffusion (M_diff_ = 0.05) compared to free diffusion (M_diff_ = 0.04), whereas intracellular diffusion (M_diff_ = 0.04) did not statistically differ from the other metrics.

For the amygdala, all diffusion metrics were significantly higher in older than younger adults, with the largest age group difference for intracellular (M_diff_ = 0.05) and dispersion (M_diff_ = 0.05) than free (M_diff_ = 0.03) diffusion.

For the nucleus accumbens, all diffusion metrics were significantly higher in older than younger adults, with the largest age group difference for intracellular (M_diff_ = 0.07) than dispersion (M_diff_ = 0.02) and free (M_diff_ = 0.03) diffusion.

For the thalamus, intracellular (M_diff_ = 0.04) and free (M_diff_ = 0.04) diffusion were significantly higher in older than younger adults, whereas the age group difference was not significant for dispersion of diffusion (M_diff_ = >−0.01). The caudate showed the same pattern of results, albeit with larger age group differences (intracellular M_diff_ = 0.10, free M_diff_ = 0.11, dispersion M_diff_ = <0.01).

For the putamen, intracellular (M_diff_ = 0.17) and free (M_diff_ = 0.11) diffusion were significantly higher in older than younger adults, with the largest age group difference for the former. In contrast, dispersion of diffusion was significantly lower in older than younger than adults (M_diff_ = −0.02).

For the globus pallidus, intracellular diffusion was significantly higher in older than younger adults (M_diff_ = 0.03). In contrast, dispersion of diffusion was significantly lower in older than younger than adults (M_diff_ = −0.07). The age group difference was not significant for free diffusion (M_diff_ = −0.01).

### 3.2. Age group differences when controlling for normalized volume

Between-group *t*-tests (Table 5) revealed significantly smaller volume in older than younger adults in frontal (M_diff_ = 24,095.5), insular (M_diff_ = 503.9), temporal (M_diff_ = 9724.4), parietal (M_diff_ = 14,898.1), and occipital (M_diff_ = 4235.3) cortex as well as the hippocampus (M_diff_ = 344.2), nucleus accumbens (M_diff_ = 145.8), thalamus (M_diff_ = 744.6), caudate (M_diff_ = 352.3), and putamen (M_diff_ = 605.4).

Separate 2 Age Group × 3 NODDI Metric omnibus ANCOVAs (Tables 2–3), as well as the subsequent post hoc 2 Age Group x 2 Metric ANCOVAs comparing each combination of NODDI Metrics (Supplementary Tables 3–4) and post hoc between-group *t*-tests for each Metric (Table 6), controlling for normalized volume in the corresponding region revealed that the pattern of significant interactions between Age Group and Metric were identical to those reported when not controlling for normalized volume, except in the following regions. For the hippocampus, a significantly larger age group difference was now seen for intracellular (M_diff_ = 0.05), not just dispersion (M_diff_ = 0.05), compared to free diffusion (M_diff_ = 0.04). For the thalamus, a significantly larger age group difference was now seen for intracellular (M_diff_ = 0.07) compared to free (M_diff_ = 0.05) diffusion, and the age group difference for dispersion of diffusion (M_diff_ = 0.01) was now significant. Thus, accounting for regional volume primarily amplified the age effect for intracellular diffusion in these regions. For the globus pallidus, the 2 Age Group × 3 Metric interaction was no longer significant, but the post hoc 2 Age Group × 2 Metric comparisons revealed the same pattern of significant interactions.

### 3.3. Age group differences when controlling for iron content

Between-group *t*-tests for QSM (Table 7) revealed significantly higher iron in older than younger adults in frontal (M_diff_ = <0.01), temporal (M_diff_ = 0.01), and parietal (M_diff_ = 0.01) cortex as well as the putamen (M_diff_ = 0.04), but lower iron in older than younger adults in the hippocampus (M_diff_ = −0.01) and thalamus (M_diff_ = −0.02). Between-group *t*-tests for R_2_* (Table 7) revealed significantly higher iron in older than younger adults in frontal cortex (M_diff_ = 0.71), occipital cortex (M_diff_ = 1.40), nucleus accumbens (M_diff_ = 2.87), putamen (M_diff_ = 9.42), and caudate (M_diff_ = 5.40).

Separate 2 Age Group × 3 NODDI Metric omnibus ANCOVAs (Tables 2–3), as well as the subsequent post hoc 2 Age Group x 2 Metric ANCOVAs comparing each combination of NODDI Metrics (Supplementary Tables 5–8) and post hoc between-group *t*-tests for each Metric (Tables 8–9), controlling for each iron metric in the corresponding region revealed that the pattern of significant interactions between Age Group and Metric were identical to those reported when not controlling for iron content, except in the following regions. For both the hippocampus and amygdala, the magnitude of the age group difference was now equivalent for intracellular, dispersion, and free diffusion when controlling for either QSM (hippocampus M_diff_ = 0.06, 0.06, and 0.06; amygdala M_diff_ = 0.04, 0.05, and 0.04) or R_2_* (hippocampus M_diff_ = 0.04, 0.05, and 0.05; amygdala M_diff_ = 0.04, 0.05, and 0.04). This finding was primarily due to the age effect for free diffusion in these regions being amplified after accounting for regional iron content. For the globus pallidus, the 2 Age Group × 3 Metric interactions remained significant, but the post hoc 2 Age Group × 2 Metric interaction comparing dispersion and free diffusion was no longer significant when controlling for R_2_*.

## 4. Discussion

The current study aimed to identify patterns of age group differences across NODDI metrics in cortical lobar, hippocampal, and subcortical gray matter in younger and older adults and to assess the effect of regional atrophy (volume) and iron content (QSM, R_2_*) on these age effects. Results revealed eight unique patterns in the direction and magnitude of age effects across NODDI metrics for the 12 regions of interest, consistent with there being region-specific neurobiological substrates of gray matter microstructure that vary in aging. Whereas some (subtle) variations in the patterns of results across regions may reflect different sensitivities to, or severities of, common neurobiological substrates captured by one or more NODDI metrics, potentially due to the NODDI metrics being significantly correlated (Supplementary Table 9), other (more prominent) variations may reflect neurobiological substrates that are unique to select regions or NODDI metrics. Thus, our discussion focuses on three prominent patterns in which (1) cortical gray matter had predominantly higher free diffusion in older than younger adults, (2) the hippocampus and amygdala had predominantly higher dispersion of diffusion and intracellular diffusion in older than younger adults, (3) the putamen and globus pallidus had lower dispersion of diffusion in older than younger adults. These prominent patterns persisted when using standardized NODDI metrics (Supplementary Figure 1, Supplementary Tables 10–12). In addition, we found that controlling for normalized volume and iron content amplified age effects for intracellular diffusion (hippocampus, thalamus) and free diffusion (hippocampus, amygdala) in select regions, respectively. These findings are detailed below.

In cortical gray matter, all lobes showed patterns in which the magnitude of the age group difference was largest for free diffusion, statistically smaller for intracellular diffusion, and even smaller or non-significant for dispersion of diffusion. Whereas free diffusion was not included in cortex in any prior NODDI study comparing younger and older adults or adults across the lifespan, studies limited to older adults similarly found that higher free diffusion was related to older age (Anderson et al., 2024; Merluzzi et al., 2016; Yu et al., 2024), with the magnitude of this age effect appearing larger than intracellular and dispersion of diffusion, as seen here. We speculate that this observed pattern of predominantly higher free diffusion in cortical gray matter in older than younger adults may reflect microstructural atrophy due to cerebrovascular disease, such as tissue loss that results from enlargement of the perivascular (Virchow-Robin) space, narrowing of the cerebral vasculature with arteriosclerosis, and cerebrovascular lesions (ischemic, infarcts, hemorrhagic) (Wardlaw et al., 2013). In support of this view, higher free water in gray matter has been linked to blood-based biomarkers of cardiovascular health (Ji et al., 2023), with similar relationships seen between free water in white matter and risk factors for cerebrovascular disease (Berger et al., 2022; Maillard et al., 2017). Moreover, cerebrovascular lesions are commonly seen in all cortical lobes as well as the caudate, putamen, globus pallidus, and thalamus (Kwee and Kwee, 2007; Looi et al., 2009; Snowdon, 1997) and we observed similarly large age-related increases in free diffusion in the caudate and putamen. Future studies using NODDI in combination with cerebrovascular disease markers in older adults are needed to further support this interpretation.

We have some evidence that predominantly higher free diffusion in cortical gray matter in aging does not reflect macrostructural (gross) atrophy (i.e., fewer voxels with tissue content measured by lower regional volume), which may plausibly co-occur with microstructural atrophy (i.e., less tissue content within voxels measured by higher free diffusion). That is, when controlling for normalized regional volume, the pattern of age group differences across NODDI metrics remained unchanged for all cortical lobar regions. Note that the pattern of results were also unchanged when alternatively controlling for cortical thickness (Supplementary Table 13). Instead, we did find that the age group difference became largest for intracellular diffusion in the hippocampus and thalamus after controlling for normalized volume. These regions are known to be vulnerable to atrophy in aging (Fjell and Walhovd, 2010; Long et al., 2012), yet it is not clear why the presence of atrophy attenuates the age group difference in intracellular diffusion in these regions, but not in other regions that showed similarly large age group differences in volume (e.g., frontal and parietal cortex, nucleus accumbens, putamen). Importantly, we were careful to minimize partial volume effects with non-tissue voxels by limiting analyses to gray matter masks that excluded voxels with white matter (conservatively using FA > 0.15 thresholding) and cerebrospinal fluid (using the non-tissue output of a two tissue-type segmentation of the *b*=0 image). Taken together, these findings support a distinction between microstructural and macrostructural atrophy, with sub-voxel level measures of tissue diffusion properties being independent of voxel-level measures of tissue volume.

Whereas our finding of higher intracellular diffusion in cortical gray matter in older than younger adults replicated at least one prior study in adults across the lifespan (Filip et al., 2023), our mixed and attenuated age effects for dispersion of diffusion contradict prior lifespan studies that have consistently reported that it is lower in older than younger adults (Filip et al., 2023; Nazeri et al., 2015; Ota et al., 2017). A possible explanation for this discrepancy is that we used a lower intrinsic diffusivity parameter that is optimized for gray matter when estimating NODDI metrics (Fukutomi et al., 2019, 2018; Guerrero et al., 2019). Not only do the NODDI metric estimates vary at different intrinsic diffusivity values, prior work suggests that the extent of this effect differs at younger versus older ages (Guerrero et al., 2019). Additional analyses reported in Supplementary Tables 14–15 support this claim. That is, when using the default intrinsic diffusivity value of 1.7 × 10^−3^ mm^2^/s, as in prior work, we observed the same lower dispersion of diffusion in older than younger adults in frontal, parietal, and occipital lobes and an attenuation of the age effect in the temporal lobe. Because both the magnitude and direction of age effects on NODDI metrics in cortical gray matter differed at lower (current study) versus higher (prior studies, current Supplementary Tables 14–15) intrinsic diffusivity values, future studies should clearly report their NODDI parameters. A complete examination of the effects of different intrinsic diffusivity values across NODDI metrics, age groups, and brain regions is also needed to better identify “optimal” intrinsic diffusivity values and whether individualized intrinsic diffusivity values are appropriate when examining age group and regional differences, such as those of interest here.

In hippocampal gray matter, all diffusion metrics were significantly higher in older than younger adults, consistent with prior work (Franco et al., 2021; Ibrahim and Bennett, 2023; Radhakrishnan et al., 2022a, 2020; Venkatesh et al., 2021, 2020). Moreover, the magnitude of this age group difference was larger for dispersion of diffusion compared to free diffusion, whereas the age effect for intracellular diffusion did not statistically differ from the other metrics. Similarly, the amygdala showed the largest age group differences for dispersion of diffusion and intracellular diffusion compared to free diffusion. We speculate that these patterns of results may be consistent with neuroinflammation, especially in the hippocampus as a comprehensive review across multiple model systems (human, non-human primates, rodents) has shown its unique vulnerability to age-related neuroinflammation (Salas et al., 2020). Neuroinflammation refers to a collection of cellular and molecular processes designed to protect the brain from acute insults (e.g., injuries, infections, stress, or trauma)(Albrecht et al., 2016; Carson et al., 2006). One cellular process involves astrocytes and microglia assuming a defensive (activated) state, in which they proliferate (early stage) and their somas swell (late stage)(Salas et al., 2020). This protective glial response can be overwhelmed in normal aging and Alzheimer’s Disease, resulting in chronic inflammation that ultimately leads to neurodegeneration, neural dysfunction, and cognitive impairments (DiSabato et al., 2016; Salas et al., 2020). Importantly, there is evidence that NODDI, notably dispersion of diffusion and intracellular diffusion, may be sensitive to glial proliferation and swelling seen in the activated glial phenotype (Garcia-Hernandez et al., 2022; Oestreich and O’Sullivan, 2022). For example, one animal study found that higher microglial density in the hippocampus of mice was related to higher dispersion of diffusion and intracellular diffusion (Yi et al., 2019). Another study using animal models of neuroinflammation found that histology-confirmed increases in glial process density were associated with higher intracellular diffusion (Garcia-Hernandez et al., 2022), whereas biological model simulations have shown that enlarged glial somas would correspond to higher dispersion of diffusion (Palombo et al., 2020). Thus, the pattern of results observed here appear to be consistent with late-stage age-related neuroinflammation in the hippocampus and possibly the amygdala (e.g., glial proliferation and swelling). In contrast, the nucleus accumbens had the largest age group difference for intracellular than dispersion and free diffusion, which may indicate earlier-stage neuroinflammation in this region (e.g., glial proliferation but not yet swelling). Future studies using NODDI in combination with other neuroinflammation markers in older adults are needed to further support this interpretation.

In dorsal striatal gray matter, very large age group differences (higher in older than younger adults) were seen for intracellular and free diffusion in the putamen and caudate, consistent with prior work (Franco et al., 2021; Guerreri et al., 2019; Venkatesh et al., 2021), whereas dispersion of diffusion was significantly lower in older than younger adults in the putamen and globus pallidus, as in prior work (Franco et al., 2021). We speculate that the latter is due to high iron content in these regions as they are known to accumulate the most iron with age (Daugherty and Raz, 2013; Ficiarà et al., 2022; Haacke et al., 2005). Iron creates inhomogeneities in the magnetic field (Gelman et al., 1999) that lead to signal loss and distortion in commonly used MRI images, including DWI (Hiwatashi et al., 2003). However, when controlling for iron content in the current study, we found that the pattern of significant age effects across NODDI metrics in the iron-rich dorsal striatal gray matter regions did not differ from those observed when not controlling for iron content, suggesting that iron content does not explain the current findings. One possible explanation for this unexpected finding is that, because the presence of iron interferes with the diffusion signal at the time of acquisition (Pfefferbaum et al., 2010), the measured signal is severely attenuated and distorted in iron-rich regions. Not only does this significantly limit our ability to accurately measure and interpret diffusion metrics in the putamen and globus pallidus, particularly in older adults, it may also result in inaccurate NODDI estimates that cannot be “corrected for” when controlling for iron content.

Strengths of the current study include our examinations of age group differences across all NODDI metrics in cortical lobar, hippocampal, and subcortical gray matter in a large sample of younger and older adults and the extent to which these age effects were driven by regional atrophy (volume) and iron content (QSM, R_2_*). We largely replicated previous reports of higher diffusion in older than younger adults in cortical (age group difference was largest for free diffusion) and hippocampal (age group difference was largest for dispersion and intracellular diffusion) gray matter, which we speculate may reflect damage due to cerebrovascular disease and neuroinflammation on gray matter microstructure in aging, respectively. We also added to an incomplete and mixed literature in subcortical gray matter by, most notably, finding lower dispersion of diffusion in older than younger adults in the putamen and globus pallidus, which did not differ when controlling for iron content, although caution is warranted when interpreting effects in these regions as the measured diffusion signal is severely attenuated and distorted by high iron content. These interpretations are limited by an absence of established markers of cerebrovascular disease and neuroinflammation that are needed to further support our interpretations and by having iron data in only a subset of the sample. Nonetheless, our observation of unique patterns in the direction and magnitude of age effects across NODDI metrics and brain regions provide valuable clues about region-specific neurobiological substrates of gray matter aging.

## Supplementary Material

1

## Figures and Tables

**Table 1:** Demographic data.

	Younger	Older	*t/χ^2^*
N	76	64	n/a
Age (years)	20.4 ± 1.9	72.5 ± 5.4	**78.8**
Sex (% female)	52.6 %	57.8 %	0.4
Education (years)	13.2 ± 1.3	15.7 ± 2.8	**6.8**
Ethnicity (% Non-Hispanic)	55.3 %	92.2 %	**23.6**
Race (% White)	30.3 %	78.1 %	**31.9**
Handedness (% right-handed)	86.8 %	82.8 %	0.4

*Notes*. Mean and standard deviation (M ± SD) or percent (%) scores are provided for each age group. Significant age group differences at *p* < 0.05 are indicated by bolded *t* (mean scores) or *χ^2^* (% scores) statistics.

**Table 2:** Omnibus ANOVA and ANCOVA results for cortical regions.

	Frontal	Insular	Temporal	Parietal	Occipital
*2 Age Group × 3 Metric ANOVA*					
Age Group	**674.6**	**739.6**	**721.7**	**410.9**	**269.5**
Metric	**3659.7**	**3176.0**	**4817.2**	**2042.5**	**2191.4**
Age Group × Metric	**399.9**	**233.0**	**287.9**	**323.9**	**248.4**
*2 Age Group × 3 Metric ANCOVA controlling for normalized volume*					
Age Group	**411.1**	**695.6**	**540.7**	**294.2**	**286.1**
Metric	**61.7**	**84.3**	**86.6**	**28.3**	**65.6**
Age Group × Metric	**302.4**	**219.0**	**209.5**	**231.8**	**252.8**
*2 Age Group × 3 Metric ANCOVA controlling for QSM*					
Age Group	**208.2**	**296.2**	**224.7**	**95.0**	**119.0**
Metric	**479.2**	**793.3**	**93.6**	**379.9**	**310.5**
Age Group × Metric	**104.8**	**108.2**	**70.8**	**68.0**	**101.9**
2 Age Group × 3 Metric ANCOVA controlling for R_2_*					
Age Group	**273.6**	**309.7**	**256.0**	**212.3**	**136.8**
Metric	**26.7**	**15.3**	**8.0**	**44.7**	**9.1**
Age Group × Metric	**185.0**	**142.9**	**101.7**	**208.4**	**109.9**

*Notes*. *F* statistics are provided for the primary omnibus ANOVA conducted in each cortical region (top row) and secondary omnibus ANCOVAs when controlling for normalized volume and iron content (QSM, R_2_*). Significant effects at *p* < 0.05 are bolded. ANCOVA results did not differ from those reported in the primary ANOVA.

**Table 3:** Omnibus ANOVA and ANCOVA results for hippocampus and subcortical regions.

	Hippocampus	Amygdala	N.Accumbens	Thalamus	Caudate	Putamen	G.Pallidus
*2 Age Group × 3 Metric ANOVA*							
Age Group	**92.4**	**65.0**	**40.7**	**14.0**	**157.8**	**173.1**	**7.4**
Metric	**2337.2**	**3024.1**	**2657.5**	**5830.8**	**1579.5**	**2544.6**	**2833.1**
Age Group × Metric	**5.4**	**5.3**	**42.3**	**27.0**	**147.2**	**324.3**	**38.0**
*2 Age Group × 3 Metric ANCOVA controlling for normalized volume*							
Age Group	**75.2**	**62.5**	**29.8**	**28.3**	**129.3**	**121.6**	**7.6**
Metric	**25.1**	**119.2**	**153.8**	**3.9**	**25.4**	**34.6**	**15.1**
Age Group × Metric	**3.5**	**6.4**	**24.2**	**39.1**	**115.0**	**228.1**	0.1
*2 Age Group × 3 Metric ANCOVA controlling for QSM*							
Age Group	**91.2**	**51.9**	**29.6**	**23.2**	**77.4**	**25.7**	0.2
Metric	**445.2**	**471.9**	**1252.4**	**1884.4**	**115.7**	**300.1**	**91.7**
Age Group × Metric	0.6	0.3	**29.3**	**21.4**	**63.4**	**10.9**	**14.3**
2 Age Group × 3 Metric controlling for R_2_*							
Age Group	**78.2**	**50.1**	**19.3**	**19.0**	**16.7**	**16.2**	0.1
Metric	**12.9**	**26.2**	**40.0**	2.9	**10.8**	**55.9**	**12.0**
Age Group × Metric	2.5	0.5	**25.1**	**24.0**	**20.0**	**32.3**	**7.6**

*Notes*. *F* statistics are provided for the primary omnibus ANOVA conducted in the hippocampus and each subcortical region (top row) and secondary omnibus ANCOVAs when controlling for normalized volume and iron content (QSM, R_2_*). Significant effects at *p* < 0.05 are bolded. ANCOVA results that differ from those reported in the primary ANOVA are underlined.

**Table 4:** Post hoc between-group *t*-test results.

	Intracellular			Dispersion			Free		
	Younger	Older	*t*	Younger	Older	*t*	Younger	Older	*t*
**Frontal**	0.22 ± 0.002	0.28 ± 0.002	**17.7**	0.44 ± 0.002	0.46 ± 0.002	**8.9**	0.30 ± 0.004	0.45 ± 0.004	**26.3**
**Insular**	0.25 ± 0.002	0.33 ± 0.002	**24.7**	0.44 ± 0.002	0.48 ± 0.002	**18.6**	0.28 ± 0.003	0.42 ± 0.003	**23.5**
**Temporal**	0.22 ± 0.001	0.26 ± 0.002	**19.1**	0.44 ± 0.001	0.46 ± 0.002	**11.5**	0.22 ± 0.002	0.34 ± 0.004	**23.8**
**Parietal**	0.22 ± 0.001	0.26 ± 0.002	**14.3**	0.44 ± 0.001	0.45 ± 0.002	**3.3**	0.29 ± 0.006	0.46 ± 0.006	**20.1**
**Occipital**	0.22 ± 0.002	0.25 ± 0.002	**10.0**	0.46 ± 0.001	0.46 ± 0.002	−0.7	0.22 ± 0.006	0.36 ± 0.006	**16.9**
**Hippocampus**	0.35 ± 0.004	0.39 ± 0.005	**7.1**	0.39 ± 0.003	0.44 ± 0.003	**12.4**	0.27 ± 0.003	0.30 ± 0.004	**7.7**
**Amygdala**	0.32 ± 0.003	0.36 ± 0.005	**8.1**	0.37 ± 0.003	0.41 ± 0.004	**8.0**	0.20 ± 0.004	0.23 ± 0.005	**5.3**
**N. Accumbens**	0.33 ± 0.005	0.40 ± 0.006	**9.5**	0.40 ± 0.003	0.42 ± 0.004	**3.7**	0.17 ± 0.006	0.20 ± 0.007	**3.3**
**Thalamus**	0.57 ± 0.007	0.61 ± 0.008	**3.7**	0.31 ± 0.003	0.31 ± 0.004	− 0.1	0.29 ± 0.004	0.33 ± 0.004	**6.2**
**Caudate**	0.37 ± 0.004	0.48 ± 0.008	**12.5**	0.41 ± 0.002	0.42 ± 0.003	0.9	0.20 ± 0.004	0.31 ± 0.007	**14.0**
**Putamen**	0.41 ± 0.006	0.58 ± 0.008	**17.5**	0.43 ± 0.002	0.41 ± 0.003	**−5.6**	0.18 ± 0.006	0.28 ± 0.007	**12.2**
**G. Pallidus**	0.65 ± 0.005	0.69 ± 0.006	**4.2**	0.34 ± 0.005	0.28 ± 0.008	**−7.1**	0.30 ± 0.005	0.29 ± 0.006	− 1.2

*Notes*. Mean ± standard error are provided for each age group and NODDI metric in each region. Significant age group differences at *p* < 0.0167 are indicated by bolded *t* statistics.

**Table 5:** Age group differences in normalized volume.

	Younger	Older	*t*
**Frontal**	128611.7 ± 17099.5	104516.2 ± 13999.8	**−9.0**
**Insular**	6771.9 ± 1055.3	6268.0 ± 961.5	**−2.9**
**Temporal**	61430.4 ± 7529.2	51706.0 ± 7364.4	**−7.7**
**Parietal**	85368.0 ± 10699.3	70469.9 ± 9179.1	**−8.7**
**Occipital**	47441.3 ± 6774.9	43205.9 ± 6883.8	**−3.6**
**Hippocampus**	3837.5 ± 37.0	3493.4 ± 46.0	**−5.9**
**Amygdala**	1220.3 ± 22.4	1265.9 ± 23.7	1.4
**N. Accumbens**	485.8 ± 11.2	340.1 ± 12.2	**−8.8**
**Thalamus**	7928.7 ± 72.8	7184.1 ± 69.2	**−7.3**
**Caudate**	3617.1 ± 48.1	3264.7 ± 37.1	**−5.6**
**Putamen**	4951.2 ± 59.9	4345.8 ± 48.5	**−7.7**
**G. Pallidus**	1648.7 ± 17.1	1628.6 ± 18.0	− 0.8

*Notes*. Mean ± standard error are provided for normalized volume in each age group and region. Significant age group differences at *p* < 0.05 are indicated by bolded *t* statistics.

**Table 6:** Post hoc between-group *F*-test results when controlling for normalized volume.

	Intracellular			Dispersion			Free		
	Younger	Older	*F*	Younger	Older	*F*	Younger	Older	*F*
**Frontal**	0.22 ± 0.002	0.27 ± 0.003	**164.4**	0.44 ± 0.002	0.46 ± 0.002	**42.3**	0.29 ± 0.004	0.45 ± 0.005	**348.5**
**Insular**	0.25 ± 0.002	0.33 ± 0.002	**304.3**	0.44 ± 0.002	0.48 ± 0.002	**172.4**	0.28 ± 0.004	0.42 ± 0.004	**273.3**
**Temporal**	0.22 ± 0.002	0.26 ± 0.002	**182.5**	0.44 ± 0.002	0.46 ± 0.002	**66.0**	0.21 ± 0.004	0.34 ± 0.004	**283.8**
**Parietal**	0.22 ± 0.002	0.26 ± 0.002	**101.8**	0.44 ± 0.001	0.45 ± 0.002	**6.4**	0.29 ± 0.006	0.46 ± 0.007	**204.5**
**Occipital**	0.22 ± 0.002	0.25 ± 0.002	**52.0**	0.46 ± 0.001	0.46 ± 0.002	0.8	0.21 ± 0.006	0.36 ± 0.006	**151.7**
**Hippocampus**	0.35 ± 0.004	0.40 ± 0.005	**25.9**	0.39 ± 0.003	0.44 ± 0.003	**76.3**	0.27 ± 0.003	0.30 ± 0.004	**29.7**
**Amygdala**	0.32 ± 0.004	0.36 ± 0.004	**34.5**	0.37 ± 0.004	0.41 ± 0.004	**33.1**	0.20 ± 0.004	0.23 ± 0.004	**15.6**
**N. Accumbens**	0.33 ± 0.006	0.40 ± 0.007	**44.8**	0.40 ± 0.004	0.42 ± 0.004	**6.9**	0.17 ± 0.007	0.20 ± 0.007	**5.7**
**Thalamus**	0.56 ± 0.007	0.62 ± 0.008	**18.1**	0.31 ± 0.004	0.32 ± 0.004	3.9	0.29 ± 0.004	0.33 ± 0.005	**22.9**
**Caudate**	0.37 ± 0.006	0.48 ± 0.007	**78.4**	0.41 ± 0.003	0.42 ± 0.003	1.0	0.20 ± 0.005	0.31 ± 0.006	**97.5**
**Putamen**	0.41 ± 0.007	0.58 ± 0.008	**152.4**	0.43 ± 0.003	0.41 ± 0.003	**15.6**	0.17 ± 0.007	0.28 ± 0.007	**74.0**
**G. Pallidus**	0.65 ± 0.005	0.69 ± 0.006	**8.9**	0.34 ± 0.006	0.28 ± 0.007	**26.0**	0.30 ± 0.005	0.29 ± 0.006	0.8

*Notes*. Mean ± standard error are provided for each age group and NODDI metric in each region when controlling for normalized volume. Significant age group differences at *p* < 0.0167 are indicated by bolded *F* statistics. Results that differ from those reported in Table 4 are underlined.

**Table 7:** Age group differences in iron content.

	QSM			R_2_*		
	Younger	Older	*t*	Younger	Older	*t*
**Frontal**	− 0.008 ± 0.001	− 0.004 ± 0.001	**3.3**	16.7 ± 0.21	17.4 ± 0.24	**2.2**
**Insular**	− 0.010 ± 0.002	− 0.015 ± 0.003	− 1.4	14.6 ± 0.20	14.7 ± 0.23	0.4
**Temporal**	0.024 ± 0.001	0.030 ± 0.001	**3.3**	20.5 ± 0.23	21.0 ± 0.27	1.4
**Parietal**	− 0.008 ± 0.001	− 0.001 ± 0.001	**5.6**	16.4 ± 0.20	16.7 ± 0.23	0.7
**Occipital**	0.013 ± 0.001	0.009 ± 0.002	− 1.6	20.0 ± 0.38	21.4 ± 0.44	**2.4**
**Hippocampus**	− 0.012 ± 0.003	− 0.026 ± 0.003	**−3.3**	15.4 ± 0.23	16.0 ± 0.43	1.3
**Amygdala**	− 0.031 ± 0.007	− 0.042 ± 0.004	− 1.4	14.1 ± 0.24	14.2 ± 0.42	0.2
**N. Accumbens**	0.020 ± 0.004	0.015 ± 0.006	− 0.8	16.6 ± 0.35	19.5 ± 0.88	**3.3**
**Thalamus**	− 0.006 ± 0.002	− 0.021 ± 0.003	**−3.9**	20.1 ± 0.20	19.9 ± 0.36	− 0.7
**Caudate**	0.048 ± 0.005	0.059 ± 0.004	1.9	19.0 ± 0.21	24.4 ± 0.70	**8.2**
**Putamen**	0.020 ± 0.002	0.062 ± 0.006	**7.5**	21.1 ± 0.27	30.6 ± 1.19	**8.6**
**G. Pallidus**	0.103 ± 0.003	0.094 ± 0.008	− 1.0	33.9 ± 0.61	35.8 ± 1.02	0.3

*Notes*. Mean ± standard error are provided for each measure of iron content (QSM, R_2_*) in each age group and region. Significant age group differences at *p* < 0.05 are indicated by bolded *t* statistics.

**Table 8:** Post hoc between-group *F*-test results when controlling for iron content (QSM).

	Intracellular			Dispersion			Free		
	Younger	Older	*F*	Younger	Older	*F*	Younger	Older	*F*
**Frontal**	0.22 ± 0.003	0.28 ± 0.003	**80.0**	0.44 ± 0.003	0.46 ± 0.003	**16.1**	0.30 ± 0.007	0.44 ± 0.008	**113.3**
**Insular**	0.25 ± 0.003	0.33 ± 0.003	**169.2**	0.44 ± 0.002	0.48 ± 0.002	**71.1**	0.28 ± 0.006	0.42 ± 0.007	**109.0**
**Temporal**	0.22 ± 0.002	0.27 ± 0.002	**119.7**	0.44 ± 0.002	0.47 ± 0.002	**38.3**	0.22 ± 0.006	0.34 ± 0.008	**82.4**
**Parietal**	0.22 ± 0.003	0.26 ± 0.003	**54.5**	0.45 ± 0.002	0.45 ± 0.003	**5.1**	0.30 ± 0.010	0.46 ± 0.012	**73.6**
**Occipital**	0.22 ± 0.002	0.25 ± 0.003	**28.7**	0.46 ± 0.002	0.46 ± 0.003	0.9	0.22 ± 0.009	0.37 ± 0.010	**64.4**
**Hippocampus**	0.34 ± 0.005	0.40 ± 0.006	**21.0**	0.38 ± 0.003	0.44 ± 0.004	**79.8**	0.25 ± 0.004	0.31 ± 0.005	**31.2**
**Amygdala**	0.31 ± 0.005	0.36 ± 0.005	**19.6**	0.37 ± 0.005	0.41 ± 0.006	**18.1**	0.19 ± 0.005	0.23 ± 0.006	**16.2**
**N. Accumbens**	0.31 ± 0.005	0.39 ± 0.006	**41.6**	0.40 ± 0.005	0.41 ± 0.006	2.2	0.15 ± 0.006	0.18 ± 0.007	3.8
**Thalamus**	0.55 ± 0.009	0.61 ± 0.011	**9.2**	0.30 ± 0.004	0.31 ± 0.004	2.4	0.27 ± 0.005	0.33 ± 0.006	**23.8**
**Caudate**	0.37 ± 0.007	0.47 ± 0.008	**49.3**	0.41 ± 0.004	0.41 ± 0.004	0.9	0.19 ± 0.008	0.29 ± 0.009	**38.3**
**Putamen**	0.43 ± 0.009	0.54 ± 0.010	**131.8**	0.43 ± 0.005	0.40 ± 0.005	**5.9**	0.18 ± 0.009	0.24 ± 0.010	**53.8**
**G. Pallidus**	0.65 ± 0.008	0.69 ± 0.009	**6.1**	0.33 ± 0.009	0.28 ± 0.010	**9.7**	0.28 ± 0.007	0.28 ± 0.008	< 0.1

*Notes*. Mean ± standard error are provided for each age group and NODDI metric in each region when controlling for QSM. Significant age group differences at *p* < 0.0167 are indicated by bolded *F* statistics. Results that differ from those reported in Table 4 are underlined.

**Table 9:** Post hoc between-group *F*-test results when controlling for iron content (R_2_*).

	Intracellular			Dispersion			Free		
	Younger	Older	*F*	Younger	Older	*F*	Younger	Older	*F*
**Frontal**	0.22 ± 0.003	0.28 ± 0.003	**80.0**	0.44 ± 0.003	0.46 ± 0.003	**16.5**	0.30 ± 0.006	0.45 ± 0.007	**148.8**
**Insular**	0.25 ± 0.003	0.33 ± 0.003	**170.4**	0.44 ± 0.002	0.48 ± 0.002	**78.9**	0.28 ± 0.006	0.42 ± 0.006	**126.7**
**Temporal**	0.22 ± 0.002	0.27 ± 0.002	**135.0**	0.44 ± 0.002	0.46 ± 0.002	**43.1**	0.22 ± 0.006	0.34 ± 0.007	**87.2**
**Parietal**	0.22 ± 0.003	0.26 ± 0.003	**54.4**	0.45 ± 0.002	0.45 ± 0.002	**8.9**	0.29 ± 0.007	0.47 ± 0.008	**125.8**
**Occipital**	0.22 ± 0.002	0.25 ± 0.003	**29.2**	0.46 ± 0.002	0.46 ± 0.003	0.9	0.22 ± 0.009	0.37 ± 0.010	**68.5**
**Hippocampus**	0.35 ± 0.005	0.39 ± 0.006	**25.0**	0.38 ± 0.003	0.44 ± 0.003	**89.5**	0.26 ± 0.004	0.31 ± 0.005	**29.2**
**Amygdala**	0.31 ± 0.005	0.36 ± 0.005	**19.8**	0.37 ± 0.005	0.41 ± 0.006	**18.1**	0.19 ± 0.005	0.23 ± 0.006	**14.7**
**N. Accumbens**	0.32 ± 0.005	0.38 ± 0.006	**45.7**	0.41 ± 0.005	0.40 ± 0.005	**6.7**	0.16 ± 0.006	0.18 ± 0.007	3.6
**Thalamus**	0.55 ± 0.009	0.60 ± 0.010	**10.1**	0.30 ± 0.004	0.31 ± 0.004	1.6	0.27 ± 0.005	0.32 ± 0.006	**19.8**
**Caudate**	0.39 ± 0.008	0.45 ± 0.009	**68.1**	0.41 ± 0.005	0.40 ± 0.006	3.7	0.20 ± 0.009	0.28 ± 0.011	**43.6**
**Putamen**	0.43 ± 0.008	0.52 ± 0.010	**175.9**	0.43 ± 0.005	0.40 ± 0.005	**5.1**	0.18 ± 0.008	0.24 ± 0.010	**65.1**
**G. Pallidus**	0.65 ± 0.008	0.68 ± 0.009	**6.0**	0.32 ± 0.008	0.29 ± 0.100	**14.4**	0.28 ± 0.007	0.28 ± 0.008	< 0.1

*Notes*. Mean ± standard error are provided for each age group and NODDI metric in each region when controlling for R_2_*. Significant age group differences at *p* < 0.0167 are indicated by bolded *F* statistics. Results that differ from those reported in Table 4 are underlined.

## Appendix A. Supporting information

Supplementary data associated with this article can be found in the online version at doi:10.1016/j.neurobiolaging.2025.02.004.
