# Supplementary material for: Aging of gray matter microstructure: A brain-wide characterization of age group differences using NODDI
Source: Neurobiol Aging. Author manuscript; Available in PMC 2026 May 21. (PMC13193764; doi:10.1016/j.neurobiolaging.2025.02.004)
Supplement: 1 [file NIHMS2171768-supplement-1.docx]

**SUPPLEMENTARY MATERIAL**

**Post Hoc 2 Age Group × 2 Metric** **ANOVA and ANCOVA**

Significant interactions were observed in the primary omnibus 2 Age Group (younger, older) × 3 NODDI Metric (intracellular, dispersion, free) mixed factorial ANOVAs for each region of interest, as well as the secondary omnibus 2 Age Group × 3 NODDI Metric ANCOVAs that controlled for regional volume (normalized volume) and iron content (QSM, R_2_^*^). Because the Metric variable has three levels, significant interactions in the omnibus ANOVAs and ANCOVAs were further probed using three post hoc 2 Age Group x 2 Metric mixed factorial ANOVAs or ANCOVAs comparing each combination of NODDI metrics (i.e., intracellular versus dispersion, intracellular versus free, dispersion versus free). Significant interactions in these post hoc ANOVAs and ANCOVAs allowed us to identify which metrics showed significant differences in the magnitude of their age group effects (e.g., when the magnitude of the age group difference was significantly larger for intracellular versus dispersion of diffusion). These post hoc analyses, in conjunction with the between-group *t*-tests for each Metric, revealed eight unique patterns in the direction and magnitude of age effects across NODDI metrics.

**Supplementary Table 1.** Post Hoc ANOVA results for the primary omnibus ANOVA in cortical regions.

|  | Frontal | Insular | Temporal | Parietal | Occipital |
| --- | --- | --- | --- | --- | --- |
| *2 Age Group* × *2 Metric (intracellular, dispersion)* | | | |  |  |
| Age Group | **211.1** | **550.1** | **260.4** | **104.7** | **26.0** |
| Metric | **53195.0** | **28481.2** | **96824.1** | **48784.7** | **109427.5** |
| Age Group × Metric | **365.5** | **350.3** | **195.3** | **295.5** | **340.3** |
| *2 Age Group* × *2 Metric (intracellular, free)* | | |  |  |  |
| Age Group | **758.1** | **713.2** | **711.8** | **436.8** | **310.7** |
| Metric | **2105.6** | **673.0** | **198.4** | **1364.2** | **180.9** |
| Age Group × Metric | **318.2** | **136.6** | **264.5** | **291.5** | **219.5** |
| *2 Age Group* × *2 Metric (dispersion, free)* | | |  |  |  |
| Age Group | **718.1** | **678.3** | **709.8** | **409.2** | **265.6** |
| Metric | **682.8** | **1592.3** | **3530.9** | **274.8** | **1429.3** |
| Age Group × Metric | **469.0** | **288.7** | **311.2** | **350.1** | **270.8** |

*Notes*. *F* statistics are provided for each post hoc ANOVA conducted in each cortical region. Significant effects at *p* < 0.05 are bolded.

**Supplementary Table 2.** Post Hoc ANOVA results for the primary omnibus ANOVA in hippocampus and subcortical regions.

|  | Hippocampus | Amygdala | N.Accumbens | Thalamus | Caudate | Putamen | G.Pallidus |
| --- | --- | --- | --- | --- | --- | --- | --- |
| *2 Age Group* × *2 Metric (intracellular, dispersion)* | | | |  |  |  |  |
| Age Group | **99.9** | **80.6** | **66.1** | **6.4** | **97.0** | **157.4** | **9.1** |
| Metric | **343.2** | **447.6** | **200.8** | **6995.3** | **7.1** | **285.8** | **2783.3** |
| Age Group × Metric | 2.7 | <0.1 | **74.8** | **34.0** | **194.1** | **496.5** | **52.2** |
| *2 Age Group* × *2 Metric (intracellular, free)* | | |  |  |  |  |  |
| Age Group | **59.4** | **48.43** | **41.8** | **23.9** | **211.4** | **248.5** | **4.1** |
| Metric | **2957.0** | **5569.0** | **5766.0** | **6461.5** | **2643.0** | **8103.6** | **6076.1** |
| Age Group × Metric | 2.8 | **16.3** | **95.5** | <0.1 | 0.1 | **117.7** | **19.1** |
| *2 Age Group* × *2 Metric (dispersion, free)* | | |  |  |  |  |  |
| Age Group | **118.1** | **56.9** | **15.6** | **12.5** | **115.1** | **65.5** | **31.4** |
| Metric | **4414.4** | **4016.9** | **3210.4** | **9.5** | **2361.2** | **2175.3** | **13.4** |
| Age Group × Metric | **10.6** | **5.2** | 1.7 | **100.1** | **239.4** | **241.6** | **30.0** |

*Notes*. *F* statistics are provided for each post hoc ANOVA conducted in the hippocampus and each subcortical region. Significant effects at *p* < 0.05 are bolded.

**Supplementary Table 3.** Post Hoc ANCOVA results for cortical regions when controlling for normalized volume.

|  | Frontal | Insular | Temporal | Parietal | Occipital |
| --- | --- | --- | --- | --- | --- |
| *2 Age Group* × *2 Metric (intracellular, dispersion)* | | | |  |  |
| Age Group | **107.3** | **520.5** | **197.4** | **72.8** | **28.5** |
| Metric | **872.6** | **779.0** | **1666.8** | **705.8** | **2586.2** |
| Age Group × Metric | **206.4** | **352.1** | **141.2** | **164.1** | **329.4** |
| *2 Age Group* × *2 Metric (intracellular, free)* | | |  |  |  |
| Age Group | **475.4** | **672.9** | **529.7** | **306.1** | **326.3** |
| Metric | **13.1** | **20.7** | 1.6 | **9.4** | <0.1 |
| Age Group × Metric | **259.5** | **124.8** | **192.6** | **218.8** | **224.2** |
| *2 Age Group* × *2 Metric (dispersion, free)* | | |  |  |  |
| Age Group | **461.2** | **633.5** | **529.9** | **303.2** | **281.0** |
| Metric | **30.0** | **39.1** | **68.9** | **10.0** | **62.2** |
| Age Group × Metric | **344.7** | **271.2** | **226.4** | **244.9** | **275.4** |

*Notes*. *F* statistics are provided for each post hoc ANCOVA conducted in each cortical region when controlling for normalized volume. Significant effects at *p* < 0.05 are bolded. Results that differ from those reported in the primary ANOVA post hoc analyses in Supplementary Table 1 are underlined.

**Supplementary Table 4.** Post Hoc ANCOVA results for hippocampus and subcortical regions when controlling for normalized volume.

|  | Hippocampus | Amygdala | N.Accumbens | Thalamus | Caudate | Putamen | G.Pallidus |
| --- | --- | --- | --- | --- | --- | --- | --- |
| *2 Age Group* × *2 Metric (intracellular, dispersion)* | | | |  |  |  |  |
| Age Group | **81.9** | **78.6** | **44.7** | **19.6** | **77.2** | **105.5** | **9.3** |
| Metric | **13.6** | **41.4** | **8.4** | **4.5** | 2.9 | 3.2 | **11.2** |
| Age Group × Metric | 0.4 | <0.1 | **44.8** | **64.2** | **143.4** | **342.2** | **53.6** |
| *2 Age Group* × *2 Metric (intracellular, free)* | | |  |  |  |  |  |
| Age Group | **50.9** | **45.7** | **30.0** | **6.6** | **169.7** | **175.8** | **4.1** |
| Metric | **11.9** | **141.5** | **354.7** | 4.7 | **50.3** | **110.0** | **37.4** |
| Age Group × Metric | **5.0** | **16.6** | **48.7** | **41.9** | 1.1 | **68.2** | **19.4** |
| *2 Age Group* × *2 Metric (dispersion, free)* | | |  |  |  |  |  |
| Age Group | **91.3** | **55.1** | **13.7** | **20.3** | **101.3** | **49.0** | **32.1** |
| Metric | **48.5** | **168.4** | **181.7** | 0.1 | **27.1** | **31.2** | 1.9 |
| Age Group × Metric | **6.4** | **7.3** | 1.9 | **72.3** | **194.0** | **183.2** | **30.6** |

*Notes*. *F* statistics are provided for each post hoc ANCOVA conducted in the hippocampus and each subcortical region when controlling for normalized volume. Significant effects at *p* < 0.05 are bolded. Results that differ from those reported in the primary ANOVA post hoc analyses in Supplementary Table 2 are underlined.

**Supplementary Table 5.** Post Hoc ANCOVA results for cortical regions when controlling for iron content (QSM).

|  | Frontal | Insular | Temporal | Parietal | Occipital |
| --- | --- | --- | --- | --- | --- |
| *2 Age Group* × *2 Metric (intracellular, dispersion)* | | | |  |  |
| Age Group | **82.4** | **283.4** | **158.8** | **32.4** | **15.4** |
| Metric | **8181.7** | **6096.1** | **1743.1** | **10599.5** | **16306.7** |
| Age Group × Metric | **133.9** | **150.6** | **63.2** | **85.4** | **159.6** |
| *2 Age Group* × *2 Metric (intracellular, free)* | | |  |  |  |
| Age Group | **224.9** | **286.1** | **188.8** | **97.5** | **133.5** |
| Metric | **295.2** | **206.3** | 1.9 | **268.0** | **38.6** |
| Age Group × Metric | **80.6** | **64.7** | **64.6** | **59.1** | **91.0** |
| *2 Age Group* × *2 Metric (dispersion, free)* | | |  |  |  |
| Age Group | **196.8** | **249.5** | **191.6** | **88.4** | **114.9** |
| Metric | **78.3** | **362.2** | **72.2** | **43.4** | **178.4** |
| Age Group × Metric | **121.7** | **130.8** | **75.8** | **74.1** | **109.4** |

*Notes*. *F* statistics are provided for each post hoc ANCOVA conducted in each cortical region when controlling for QSM. Significant effects at *p* < 0.05 are bolded. Results that differ from those reported in the primary ANOVA post hoc analyses in Supplementary Table 1 are underlined.

**Supplementary Table 6.** Post Hoc ANCOVA results for hippocampus and subcortical regions when controlling for iron content (QSM).

|  | Hippocampus | Amygdala | N.Accumbens | Thalamus | Caudate | Putamen | G.Pallidus |
| --- | --- | --- | --- | --- | --- | --- | --- |
| *2 Age Group* × *2 Metric (intracellular, dispersion)* | | | |  |  |  |  |
| Age Group | **91.3** | **50.4** | **41.9** | **13.8** | **53.5** | **20.4** | 0.3 |
| Metric | **53.3** | **82.9** | **114.1** | **1931.1** | <0.1 | 3.6 | **68.2** |
| Age Group × Metric | 0.8 | 0.4 | **53.9** | **25.3** | **120.7** | **85.3** | **20.9** |
| *2 Age Group* × *2 Metric (intracellular, free)* | | | |  |  |  |  |
| Age Group | **56.7** | **39.8** | **34.7** | **29.6** | **999.7** | **39.9** | **5.0** |
| Metric | **582.4** | **895.8** | **2626.6** | **2597.3** | **197.7** | **849.1** | **264.0** |
| Age Group × Metric | <0.1 | <0.1 | **70.7** | 0.5 | <0.1 | **15.5** | **9.3** |
| *2 Age Group* × *2 Metric (dispersion, free)* | | |  |  |  |  |  |
| Age Group | **126.6** | **49.5** | **8.5** | **25.9** | **53.1** | **6.3** | **7.5** |
| Metric | **778.6** | **622.4** | **1469.1** | **4.6** | **128.7** | **286.3** | **11.7** |
| Age Group × Metric | 0.8 | 0.2 | 0.6 | **60.4** | **72.0** | **27.3** | **8.5** |

*Notes*. *F* statistics are provided for each post hoc ANCOVA conducted in the hippocampus and each subcortical region when controlling for QSM. Significant effects at *p* < 0.05 are bolded. Results that differ from those reported in the primary ANOVA post hoc analyses in Supplementary Table 2 are underlined.

**Supplementary Table 7.** Post Hoc ANCOVA results for cortical regions when controlling for iron content (R_2_^*^).

|  | Frontal | Insular | Temporal | Parietal | Occipital |
| --- | --- | --- | --- | --- | --- |
| *2 Age Group* × *2 Metric (intracellular, dispersion)* | | | |  |  |
| Age Group | **67.5** | **240.4** | **179.4** | **59.3** | **9.5** |
| Metric | **97.5** | **34.5** | **124.1** | **49.2** | **491.5** |
| Age Group × Metric | **154.4** | **190.1** | **81.1** | **184.1** | **16.0** |
| *2 Age Group* × *2 Metric (intracellular, free)* | | |  |  |  |
| Age Group | **316.9** | **317.5** | **224.8** | **242.4** | **150.3** |
| Metric | **43.5** | **26.2** | **9.5** | **62.4** | **5.4** |
| Age Group × Metric | **154.4** | **79.4** | **95.0** | **180.7** | **99.5** |
| *2 Age Group* × *2 Metric (dispersion, free)* | | |  |  |  |
| Age Group | **286.6** | **261.7** | **224.8** | **172.3** | **132.5** |
| Metric | **7.7** | 3.9 | <0.1 | **30.1** | 1.5 |
| Age Group × Metric | **210.7** | **182.3** | **107.8** | **232.2** | **117.3** |

*Notes*. *F* statistics are provided for each post hoc ANCOVA conducted in each cortical region when controlling for R_2_^*^. Significant effects at *p* < 0.05 are bolded. Results that differ from those reported in the primary ANOVA post hoc analyses in Supplementary Table 1 are underlined.

**Supplementary Table 8.** Post Hoc ANCOVA results for hippocampus and subcortical regions when controlling for iron content (R_2_^*^).

|  | Hippocampus | Amygdala | N.Accumbens | Thalamus | Caudate | Putamen | G.Pallidus |
| --- | --- | --- | --- | --- | --- | --- | --- |
| *2 Age Group* × *2 Metric (intracellular, dispersion)* | | | |  |  |  |  |
| Age Group | **82.5** | **50.8** | **27.6** | **11.0** | **7.3** | **12.9** | 0.2 |
| Metric | **10.6** | **6.2** | **4.7** | 3.5 | **5.5** | **15.9** | 0.9 |
| Age Group × Metric | 4.0 | 0.5 | **47.6** | **26.4** | **27.8** | **70.2** | **11.6** |
| *2 Age Group* × *2 Metric (intracellular, free)* | | |  |  |  |  |  |
| Age Group | **43.7** | **37.0** | **25.0** | **25.7** | **25.3** | **27.6** | 3.9 |
| Metric | 2.9 | **43.1** | **83.9** | 3.3 | **7.1** | **69.6** | **32.1** |
| Age Group × Metric | 0.6 | 0.1 | **51.6** | 0.3 | 1.7 | **12.1** | **6.6** |
| *2 Age Group* × *2 Metric (dispersion, free)* | | |  |  |  |  |  |
| Age Group | **113.1** | **47.2** | **4.2** | **19.9** | **11.0** | 2.8 | 2.6 |
| Metric | **21.5** | **35.0** | **48.5** | 0.4 | **16.7** | **74.3** | **12.9** |
| Age Group × Metric | 2.0 | 1.3 | 2.1 | **64.9** | **27.8** | **11.3** | 3.1 |

*Notes*. *F* statistics are provided for each post hoc ANCOVA conducted in the hippocampus and each subcortical region when controlling for R_2_^*^. Significant effects at *p* < 0.05 are bolded. Results that differ from those reported in the primary ANOVA post hoc analyses in Supplementary Table 2 are underlined.

**Correlations Among NODDI Metrics**

Relationships among NODDI metrics were assessed using three separate partial correlations in each region of interest, controlling for age group. Results revealed significant relationships among all NODDI metrics, with the exception of the relationship between dispersion of diffusion and free diffusion in all cortical regions except insular cortex.

**Supplementary Table 9.** Partial correlations among NODDI metrics.

|  | Intracellular × Dispersion | Intracellular × Free | Dispersion × Free |
| --- | --- | --- | --- |
| Frontal | **0.83** | **0.34** | 0.09 |
| Insular | **0.78** | **0.57** | **0.31** |
| Temporal | **0.82** | **0.25** | -0.06 |
| Parietal | **0.74** | **0.42** | 0.01 |
| Occipital | **0.83** | **0.26** | -0.02 |
| Hippocampus | **0.70** | **0.84** | **0.63** |
| Amygdala | **0.63** | **0.83** | **0.54** |
| N. Accumbens | **0.57** | **0.85** | **0.44** |
| Thalamus | **0.86** | **0.76** | **0.79** |
| Caudate | **0.51** | **0.64** | **0.51** |
| Putamen | **0.53** | **0.79** | **0.40** |
| G. Pallidus | *-0.25* | *0.21* | *0.26* |

*Notes*. Correlation coefficients are provided for each partial correlation among NODDI metric that controlled for age group in each region. Significant relationships at *p <* 0.001 or *p <* 0.05 are indicated by bolded or italicized *r* statistics, respectively.

**Standardized NODDI Metrics**

Because the NODDI metrics are on different scales of measurement, age group differences in gray matter microstructure were re-assessed after standardizing scores (z-score) using the mean and standard deviation across all participants and regions for each metric. When using standardizing scores, separate 2 Age Group × 3 NODDI Metric omnibus ANOVAs revealed significant interactions for each cortical (Supplementary Table 10, top row) and subcortical (Supplementary Table 11, top row) region of interest, except the nucleus accumbens. Post hoc 2 Age Group x 2 Metric ANOVAs comparing each combination of NODDI metrics for each region (Supplementary Tables 10-11) and post hoc between-group *t*-tests for each Metric (Supplementary Table 12) revealed nine unique patterns in the direction and magnitude of age effects across NODDI metrics, as detailed below and summarized in Supplementary Figure 1.

For frontal cortex, all diffusion metrics were significantly higher in older than younger adults, with the largest age group difference for free (mean difference: M_diff_ = 1.72) compared to intracellular (M_diff_ = 0.37) and dispersion of diffusion (M_diff_ = 0.37).

For insular cortex, all diffusion metrics were significantly higher in older than younger adults, with the largest age group difference for free (M_diff_ = 1.56), then dispersion (M_diff_ = 0.74), and then intracellular (M_diff_ = 0.54) diffusion. Temporal cortex showed the same pattern of results (free M_diff_ = 1.45, dispersion M_diff_ = 0.43, intracellular M_diff_ = 0.29).

For parietal cortex, all diffusion metrics were significantly higher in older than younger adults, with the largest age group difference for free (M_diff_ = 1.90), then intracellular (M_diff_ = 0.26), and then dispersion of diffusion (M_diff_ = 0.12).

For occipital cortex, free (M_diff_ = 1.65) and intracellular (M_diff_ = 0.15) diffusion were significantly higher in older than younger adults, with the largest age group difference for the former, whereas there was no significant age effect for dispersion of diffusion (M_diff_ = 0.03). Thalamus (free M_diff_ = 0.42, intracellular M_diff_ = 0.26, dispersion M_diff_ = 0.02), and caudate (free M_diff_ = 1.24, intracellular M_diff_ = 0.70, dispersion M_diff_ = 0.06) showed the same pattern of results.

For the hippocampus, all diffusion metrics were significantly higher in older than younger adults, with the largest age group difference for dispersion of diffusion (M_diff_ = 0.84), then free diffusion (M_diff_ = 0.43), and then intracellular diffusion (M_diff_ = 0.29).

For the amygdala, all diffusion metrics were significantly higher in older than younger adults, with the largest age group difference for dispersion of diffusion (M_diff_ = 0.78) compared to free (M_diff_ = 0.37) and intracellular (M_diff_ = 0.31) diffusion.

For the nucleus accumbens, all diffusion metrics were significantly higher in older than younger adults, with comparable age group differences for intracellular (M_diff_ = 0.50), free (M_diff_ = 0.33), and dispersion of diffusion (M_diff_ = 0.32).

For the putamen, free (M_diff_ = 1.23) and intracellular (M_diff_ = 1.15) diffusion were significantly higher in older than younger adults. In contrast, dispersion of diffusion was significantly lower in older than younger than adults (M_diff_ = -0.36).

For the globus pallidus, intracellular diffusion was significantly higher in older than younger adults (M_diff_ = 0.23). In contrast, dispersion of diffusion was significantly lower in older than younger than adults (M_diff_ = -1.15). The age group difference was not significant for free diffusion (M_diff_ = -0.11).

Taken together, although the results revealed nine unique patterns in the direction and magnitude of age effects across NODDI metrics that differed slightly from those seen when not standardizing scores (e.g., frontal cortex now showed a slightly different pattern of results relative to insular, temporal, and parietal cortices), the three prominent patterns detailed in the Discussion section remained unchanged (i.e., cortical regions had predominantly higher free diffusion in older than younger adults, the hippocampus and amygdala had predominantly higher dispersion of diffusion in older than younger adults, and the putamen and globus pallidus had lower dispersion of diffusion in older than younger adults; see Supplementary Figure 1).

**Supplementary Figure 1.** Age group differences between younger (Y) and older (O) adults are summarized for each NODDI metric and gray matter region of interest from analyses using standardized NODDI metrics (z-scores). Arrows indicate patterns of relative age group differences (>>> significantly largest, >> significantly larger, > significant, = not significant). Colors provide a heatmap of the magnitude of age group differences.

**Supplementary Table 10.** ANOVA results for cortical regions when using standardized scores.

|  | Frontal | Insular | Temporal | Parietal | Occipital |
| --- | --- | --- | --- | --- | --- |
| *Omnibus 2 Age Group* × *3 Metric (intracellular, dispersion, free)* | | | | | |
| Age Group | **642.5** | **725.9** | **704.2** | **393.2** | **245.7** |
| Metric | **2392.7** | **1846.6** | **1553.0** | **1397.1** | **879.4** |
| Age Group × Metric | **370.5** | **212.2** | **257.9** | **315.2** | **245.3** |
| *Post Hoc 2 Age Group* × *2 Metric (intracellular, dispersion)* | | | | | |
| Age Group | **152.1** | **479.5** | **202.0** | **55.0** | **6.5** |
| Metric | **12565.9** | **11826.8** | **13767.8** | **13358.9** | **17758.6** |
| Age Group × Metric | < 0.1 | **56.4** | **27.5** | **29.3** | **49.2** |
| *Post Hoc 2 Age Group* × *2 Metric (intracellular, free)* | | | | | |
| Age Group | **761.1** | **673.2** | **410.7** | **430.5** | **304.6** |
| Metric | **3783.9** | **2218.6** | **833.0** | **2054.4** | **464.2** |
| Age Group × Metric | **498.3** | **320.7** | **396.5** | **348.0** | **251.0** |
| *Post Hoc 2 Age Group* × *2 Metric (dispersion, free)* | | | | | |
| Age Group | **668.9** | **694.1** | **709.8** | **394.0** | **244.7** |
| Metric | **90.0** | **5.7** | **386.9** | **82.6** | **184.9** |
| Age Group × Metric | **334.1** | **155.8** | **199.0** | **310.0** | **252.7** |

*Notes*. *F* statistics are provided for each omnibus (top row) and post hoc ANOVA conducted in each cortical region when using standardized scores. Significant effects at *p* < 0.05 are bolded. Results that differ from those reported in the primary ANOVA (Table 2) and its post hoc analyses (Supplementary Table 1) are underlined.

**Supplementary Table 11.** ANOVA results for hippocampus and subcortical regions when using standardized scores.

|  | Hippocampus | Amygdala | N.Accumbens | | Thalamus | | Caudate | | Putamen | | G.Pallidus | |
| --- | --- | --- | --- | --- | --- | --- | --- | --- | --- | --- | --- | --- |
| *Omnibus 2 Age Group* × *3 Metric (intracellular, dispersion, free)* | | | | |  | |  | |  | |  | |
| Age Group | **112.8** | **66.6** | **31.2** | | **10.2** | | **121.6** | | **106.7** | | **25.6** | |
| Metric | 2.3 | **136.6** | **483.4** | | **8284.0** | | **184.0** | | **673.6** | | **1240.0** | |
| Age Group × Metric | **84.5** | **26.5** | 3.3 | | **39.0** | | **132.3** | | **270.1** | | **44.9** | |
| *Post Hoc 2 Age Group* × *2 Metric (intracellular, dispersion)* | | | |  | |  | |  | |  | |  |
| Age Group | **127.5** | **76.6** | - | | 2.6 | | **47.5** | | **49.6** | | **34.1** | |
| Metric | 3.0 | **7.9** | - | | **19268.7** | | **64.1** | | **380.3** | | **1681.1** | |
| Age Group × Metric | **129.4** | **35.8** | - | | **34.3** | | **95.0** | | **524.5** | | **55.8** | |
| *Post Hoc 2 Age Group* × *2 Metric (intracellular, free)* | | |  | |  | |  | |  | |  | |
| Age Group | **60.6** | **42.9** | - | | **27.8** | | **215.4** | | **224.6** | | 1.2 | |
| Metric | **7.8** | **665.8** | - | | **2305.6** | | **418.6** | | **2072.4** | | **1737.9** | |
| Age Group × Metric | **21.6** | 1.9 | - | | **12.3** | | **62.2** | | 1.7 | | **13.1** | |
| *Post Hoc 2 Age Group* × *2 Metric (dispersion, free)* | | |  | |  | |  | |  | |  | |
| Age Group | **130.0** | **61.4** | - | | **8.0** | | **87.2** | | **39.6** | | **38.3** | |
| Metric | < 0.1 | **112.2** | - | | **5328.4** | | **104.0** | | **246.3** | | **524.2** | |
| Age Group × Metric | **64.6** | **24.6** | - | | **62.8** | | **202.6** | | **262.6** | | **40.9** | |

*Notes*. *F* statistics are provided for each omnibus (top row) and post hoc ANOVA conducted in each cortical region when using standardized scores. Significant effects at *p* < 0.05 are bolded. Post hoc ANOVAs were not performed for the nucleus accumbens given that the interaction was not significant in the omnibus ANOVA. Results that differ from those reported in the primary ANOVA (Table 3) and its post hoc analyses (Supplementary Table 2) are underlined.

**Supplementary Table 12.** Post hoc between-group *t*-test results when using standardized scores.

|  | Intracellular | | | Dispersion | | | Free | | |
| --- | --- | --- | --- | --- | --- | --- | --- | --- | --- |
|  | Younger | Older | *t* | Younger | Older | *t* | Younger | Older | *t* |
| Frontal | -1.02 ± 0.01 | -0.65 ± 0.02 | **17.7** | 0.50 ± 0.03 | 0.87 ± 0.03 | **8.9** | 0.17 ± 0.05 | 1.90 ± 0.05 | **26.3** |
| Insular | -0.86 ± 0.01 | -0.32 ± 0.02 | **24.7** | 0.47 ± 0.03 | 1.21 ± 0.03 | **18.6** | -0.02 ± 0.04 | 1.54 ± 0.05 | **23.5** |
| Temporal | -1.03 ± 0.01 | -0.74 ± 0.01 | **19.1** | 0.45 ± 0.03 | 0.88 ± 0.03 | **11.5** | -0.77 ± 0.03 | 0.68 ± 0.06 | **23.8** |
| Parietal | -1.02 ± 0.01 | -0.76 ± 0.02 | **14.3** | 0.57 ± 0.02 | 0.69 ± 0.03 | **3.3** | 0.14 ± 0.06 | 2.04 ± 0.07 | **20.1** |
| Occipital | -0.86 ± 0.01 | -1.01 ± 0.01 | **10.0** | 0.82 ± 0.02 | 0.79 ± 0.03 | -0.7 | -0.74 ± 0.05 | 0.91 ± 0.09 | **16.9** |
| Hippocampus | -0.14 ± 0.03 | 0.15 ± 0.03 | **7.1** | -0.40 ± 0.05 | 0.48 ± 0.06 | **12.4** | -0.17 ± 0.04 | 0.26 ± 0.04 | **7.7** |
| Amygdala | -0.37 ± 0.02 | -0.06 ± 0.03 | **8.1** | -0.72 ± 0.06 | 0.06 ± 0.08 | **8.0** | -0.96 ± 0.04 | -0.59 ± 0.05 | **5.3** |
| N. Accumbens | -0.32 ± 0.03 | 0.18 ± 0.04 | **9.5** | -0.19 ± 0.05 | 0.13 ± 0.08 | **3.7** | -1.29 ± 0.07 | -0.96 ± 0.08 | **3.3** |
| Thalamus | 1.33 ± 0.05 | 1.59 ± 0.05 | **3.7** | -1.68 ± 0.06 | -1.70 ± 0.06 | -0.1 | 0.10 ± 0.05 | 0.52 ± 0.05 | **6.2** |
| Caudate | 0.00 ± 0.03 | 0.70 ± 0.05 | **12.5** | 0.06 ± 0.04 | 0.12 ± 0.06 | 0.9 | -0.95 ± 0.05 | 0.29 ± 0.08 | **14.0** |
| Putamen | 0.23 ± 0.04 | 1.38 ± 0.05 | **17.5** | 0.34 ± 0.04 | -0.02 ± 0.05 | **-5.6** | -1.22 ± 0.06 | 0.01 ± 0.08 | **12.2** |
| G. Pallidus | 1.90 ± 0.04 | 2.13 ± 0.04 | **4.2** | -1.20 ± 0.10 | -2.35 ± 0.14 | **-7.1** | 0.16 ± 0.06 | 0.05 ± 0.07 | -1.2 |

*Notes*. Mean ± standard error are provided for each age group and NODDI metric in each region when using standardized scores. Significant age group differences at *p <* 0.0167 are indicated by bolded *t* statistics. Results did not differ from those reported in the primary ANOVA post hoc analyses in Table 4.

**Results when Controlling for Cortical Thickness**

Cortical thickness was estimated in each cortical region of interest and in each hemisphere for each participant using FreeSurfer (Version 7.1.1) *mri_segstats* and then averaged across hemispheres. Here we report age group differences in cortical gray matter when controlling for cortical thickness in the corresponding region. Results revealed that the pattern of age group differences were identical to those reported when controlling for normalized volume, both of which were identical to those reported when not controlling for any measure of macrostructural atrophy.

**Supplementary Table 13.** ANCOVA results for cortical regions when controlling for cortical thickness.

|  | Frontal | Insular | Temporal | Parietal | Occipital |
| --- | --- | --- | --- | --- | --- |
| *Omnibus 2 Age Group* × *3 Metric (intracellular, dispersion, free)* | | | |  |  |
| Age Group | **233.5** | **412.3** | **331.2** | **183.2** | **133.6** |
| Metric | **7.2** | **9.3** | **23.0** | **6.0** | **8.0** |
| Age Group × Metric | **128.8** | **151.2** | **176.0** | **167.2** | **183.4** |
| *Post Hoc 2 Age Group* × *2 Metric (intracellular, dispersion)* | | | |  |  |
| Age Group | **55.9** | **266.2** | **107.7** | **25.5** | **5.7** |
| Metric | **77.7** | **53.7** | **190.5** | **76.8** | **246.3** |
| Age Group × Metric | **266.0** | **236.8** | **285.7** | **282.4** | **412.6** |
| *Post Hoc 2 Age Group* × *2 Metric (intracellular, free)* | | |  |  |  |
| Age Group | **306.2** | **440.4** | **421.3** | **233.3** | **203.1** |
| Metric | 1.4 | 0.5 | 0.5 | **6.4** | 1.1 |
| Age Group × Metric | **62.9** | **69.6** | **110.3** | **113.6** | **129.6** |
| *Post Hoc 2 Age Group* × *2 Metric (dispersion, free)* | | |  |  |  |
| Age Group | **213.6** | **385.7** | **323.0** | **163.5** | **132.3** |
| Metric | 3.8 | **7.0** | **24.8** | 0.1 | **4.6** |
| Age Group × Metric | **168.8** | **194.2** | **218.8** | **199.8** | **223.2** |

*Notes*. *F* statistics are provided for each omnibus (top row) and post hoc ANOVA conducted in each cortical region when controlling for cortical thickness. Significant effects at *p* < 0.05 are bolded. Results that differ from those reported in the primary ANOVA (Table 2) and its post hoc analyses (Supplementary Table 1) are underlined.

**Results for Intrinsic Diffusivity of 1.7 × 10^−3^ mm^2^/s**

The pattern of age effects in cortical gray matter observed in the current manuscript when using an intrinsic diffusivity value of 1.1 × 10^−3^ mm^2^/s to more accurately model the tissue compartments in gray matter (Fukutomi et al., 2018, 2019; Guerrero et al., 2019) differed from those reported in prior NODDI studies in adults across the lifespan (Filip et al., 2023; Nazeri et al., 2015; Ota et al., 2017) and within older adults (Anderson et al., 2024; Merluzzi et al., 2016; Yu et al., 2024) that used the default intrinsic diffusivity value of 1.7 × 10^−3^ mm^2^/s. Here we report age group differences in cortical gray matter when using the default intrinsic diffusivity value to demonstrate that this parameter influences the magnitude and direction of age group differences for the dispersion of diffusion and intracellular diffusion metrics.

**Supplementary Table 14.** ANOVA results for cortical regions when using the default intrinsic diffusivity value of 1.7 × 10^−3^ mm^2^/s.

|  | Frontal | Insular | Temporal | Parietal | Occipital |
| --- | --- | --- | --- | --- | --- |
| *Omnibus 2 Age Group* × *3 Metric (intracellular, dispersion, free)* | | | | | |
| Age Group | **561.7** | **724.8** | **663.0** | **349.2** | **203.7** |
| Metric | **6950.4** | **7542.5** | **13373.9** | **3690.5** | **5358.0** |
| Age Group × Metric | **483.3** | **350.0** | **406.4** | **362.5** | **260.6** |
| *Post Hoc 2 Age Group* × *2 Metric (intracellular, dispersion)* | | | | | |
| Age Group | **19.6** | **345.4** | **4.4** | **11.6** | **62.3** |
| Metric | **59137.2** | **38733.3** | **118850.3** | **63658.4** | **79717.1** |
| Age Group × Metric | **169.5** | **357.9** | **15.6** | **64.8** | 2.1 |
| *Post Hoc 2 Age Group* × *2 Metric (intracellular, free)* | | | | | |
| Age Group | **650.3** | **707.9** | **706.9** | **401.9** | **269.4** |
| Metric | **278.7** | **1212.0** | **2684.5** | **92.1** | **835.2** |
| Age Group × Metric | **411.5** | **253.6** | **380.3** | **337.9** | **240.4** |
| *Post Hoc 2 Age Group* × *2 Metric (dispersion, free)* | | | | | |
| Age Group | **617.0** | **634.6** | **709.8** | **383.7** | **237.9** |
| Metric | **8483.2** | **9337.7** | **18952.9** | **4288.1** | **7742.5** |
| Age Group × Metric | **584.3** | **428.3** | **457.8** | **401.4** | **294.9** |

*Notes*. *F* statistics are provided for each omnibus (top row) and post hoc ANOVA conducted in each cortical region. Significant effects at *p* < 0.05 are bolded. Results that differ from those reported in the primary ANOVA (Table 2) and its post hoc analyses (Supplementary Table 1) are underlined.

**Supplementary Table 15.** Post hoc between-group *t*-test results when using the default intrinsic diffusivity value of 1.7 × 10^−3^ mm^2^/s.

|  | Intracellular | | | Dispersion | | | Free | | |
| --- | --- | --- | --- | --- | --- | --- | --- | --- | --- |
|  | Younger | Older | *t* | Younger | Older | *t* | Younger | Older | *t* |
| Frontal | 0.33 ± 0.002 | 0.35 ± 0.002 | **8.0** | 0.60 ± 0.001 | 0.59 ± 0.001 | **-2.9** | 0.20 ± 0.004 | 0.36 ± 0.005 | **25.2** |
| Insular | 0.35 ± 0.002 | 0.41 ± 0.002 | **20.9** | 0.59 ± 0.001 | 0.60 ± 0.001 | **9.5** | 0.19 ± 0.004 | 0.35 ± 0.005 | **23.6** |
| Temporal | 0.35 ± 0.002 | 0.35 ± 0.002 | **2.9** | 0.60 ± 0.001 | 0.60 ± 0.001 | 0.6 | 0.12 ± 0.004 | 0.25 ± 0.004 | **24.6** |
| Parietal | 0.33 ± 0.002 | 0.33 ± 0.002 | 0.6 | 0.60 ± 0.001 | 0.59 ± 0.001 | **-9.3** | 0.20 ± 0.006 | 0.37 ± 0.006 | **20.1** |
| Occipital | 0.35 ± 0.002 | 0.33 ± 0.002 | **-5.4** | 0.62 ± 0.001 | 0.60 ± 0.002 | **-11.3** | 0.11 ± 0.006 | 0.26 ± 0.007 | **16.6** |

*Notes*. Mean ± standard error are provided for each age group and NODDI metric in each region. Significant age group differences at *p <* 0.0167 are indicated by bolded *t* statistics. Results that differ from those reported in the primary ANOVA post hoc analyses in Table 4 in term of direction or significance are underlined.
